# Municipal resources and patient outcomes through the first year after a hip fracture

**DOI:** 10.1186/s12913-017-2087-5

**Published:** 2017-02-16

**Authors:** Sabine Ruths, Valborg Baste, Marit Stordal Bakken, Lars Birger Engesæter, Stein Atle Lie, Siren Haugland

**Affiliations:** 1 Research Unit for General Practice, Uni Research Health, Bergen, Norway; 2 Department of Global Public Health and Primary Care, University of Bergen, Kalfarveien 31, N-5018 Bergen, Norway; 3 Uni Research Health, Bergen, Norway; 4 Department of Clinical Medicine, University of Bergen, Bergen, Norway; 5 Norwegian Arthroplasty Register, Haukeland University Hospital, Bergen, Norway; 6 Department of Clinical Dentistry, University of Bergen, Bergen, Norway

**Keywords:** Hip fracture, Survival, Quality of life, Municipal resources, Health services

## Abstract

**Background:**

Hip fractures represent major critical events for older people, and put huge demands on economic and personnel resources. Most hip fracture patients are in need of postoperative rehabilitation services. Through the Coordination Reform, the municipalities in Norway were given increased responsibility for community-based treatment and rehabilitation after surgery. The purpose of this study was to examine associations between municipal resources and patient outcomes through the first year after a hip fracture, focusing on survival and health-related quality of life.

**Methods:**

We conducted a nationwide cohort study on people experiencing a hip fracture in 2011–2012 in Norway, with a 1-year follow-up. We obtained data on date of hip fracture, demographics, total morbidity (ASA) score, health-related quality of life (EQ-5D-3 L), date of death if applicable, municipality of residence (Norwegian Hip Fracture Register), date of hospital readmission due to complications (Norwegian Patient Register), and information on municipalities’ characteristics (Municipality-State-Reporting).

**Results:**

The study population comprised 15,757 patients, mean age 80.8 years, 68.6% women. All-cause mortality was 8.6% at 30 days, and 25.3% at 12 months. Mortality was lower in the municipalities with the highest overall staff time for rehabilitation. A high proportion of the population aged 80+, was associated with low rates of self-reported anxiety/depression 12 months after surgery, as well as higher general health scores (EQ-5D VAS). There were no other differences in outcome according to rehabilitation resources, when comparing municipalities with the highest and lowest staffing.

**Conclusion:**

The study revealed no substantial impact of municipal resources on survival and health-related quality of life through the first year after a hip fracture. To evaluate major organizational changes and allocate resources according to best practice, there is a need to monitor health outcomes and use of resources over time through reliable measures, including variables related to coordination between services.

**Electronic supplementary material:**

The online version of this article (doi:10.1186/s12913-017-2087-5) contains supplementary material, which is available to authorized users.

## Background

Hip fractures represent critical events for older people with complex health problems, often resulting in loss of function and physical disability, nursing home admittance or early death [1–3]. The incidence of hip fractures increases exponentially with age, and is higher in Norway than in most other countries [4]. Due to demographic changes, the number of hip fractures is expected to increase dramatically [5]. Studies from European countries indicate that hip fractures are the cause of 8% of emergency department visits, and 42% of fall-related hospital admissions [6, 7].

As there are large differences with regard to available health care services, it is of interest to examine municipal resources in relation to patient outcomes. Patients with hip fracture constitute a group of particular interest, due to the high incidence, defined time of onset, relatively well-defined rehabilitation needs within a certain time frame, and the rehabilitation potential. Although hospital based treatment is crucial for short- and long-term outcomes after such fractures, there is an increased need to document use of community based health services. Previous research shows that increased efforts for rehabilitation and continuity of care may reduce the total care costs after hip fractures [8]. According to a study in Norway, half of the hip fracture patients received rehabilitation provided by municipal home services (17%) or nursing home (short-term stay, 33%) [9]. A comparative study indicated that structured inpatient rehabilitation centres may improve functional ability and reduce the need for care among citizens 65 years and older, compared to standard primary health care rehabilitation [10]. There is also evidence that multidisciplinary home based rehabilitation may improve patient outcomes [11].

In many countries, decision makers and planners have promoted coordination of health services to improve quality and reduce costs. In Norway, *The Coordination Reform* [12] has been gradually implemented in the period 2012 to 2016, targeting municipal health services for older patients with complex health problems. Through this reform, the municipalities were given increased responsibility for community-based treatment and rehabilitation (“lowest effective care level”) for patients after hospital stays. The national aims are to offer adequate staffing capacity and multidisciplinary competence in primary health care, as well as planned patient trajectories and committed cooperation with hospitals [13].

To shed light on the context of the Coordination reform, the present study aimed to examine associations between municipal resources and patient outcomes through the first year after a hip fracture, focusing on survival and health-related quality of life.

## Methods

### Design

We conducted a nationwide cohort study on hip fractures occurring in the period from 1 January 2011 to 31 December 2012, based on data from the Norwegian Hip Fracture Register (NHFR) [14], with 1-year follow-up. The cohort was merged with the Norwegian Patient Register (NPR) at the Norwegian Directorate of Health [15] by personal identification, and municipality resources were incorporated from the Municipality-State-Reporting (KOSTRA) at Statistics Norway [16].

### Data sources

The Norwegian Hip Fracture Register contains information on patients operated for hip fracture from 2005 at all hospitals in Norway that perform these operations [14]. All hip fractures reported to NHFR in the study period were included. Variables extracted for this study comprised the date of surgery, patients’ age and gender, municipality of residence, preoperative ASA (American Society of Anesthesiologists) score, health-related quality of life (EQ-5D-3 L) preoperative (reported 4 month postoperative) and at 12 months postoperative, and in case of death within 1 year after operation, the date of death. The EQ-5D-3 L is a descriptive tool assessing 5 dimensions: the level of mobility, self-care, usual activity, pain/discomfort, and anxiety/depression [17]. Each question has 3 levels: no problems, some problems, and severe problems. A visual analog scale (VAS) described how satisfied the patients were with their general health condition. The value 0 represented very dissatisfied while the value 100 represented very satisfied.

NHFR data was merged with data in the Norwegian Patient Register (NPR) using the unique 11-digit personal identity number assigned to all residents of Norway. NPR is a national health register containing information on all patients who have received specialist health services from 1996 [15]. We extracted data from NPR regarding length of hospital stay for primary hip fracture surgery (date of admission and discharge), and hospital readmissions during the first year post surgery due to complications such as bleeding, wound infection, mechanical problem, or venous thromboembolic complications (ICD-10 diagnostic codes T81 and T84; date of admission and discharge) [18].

Municipality-State-Reporting (KOSTRA) is a national information system that contains management information on municipal key activities including health services from 1995 [16]. Administrative data (e.g. patients, staffing and costs) are collected quarterly from all 428 municipalities. We extracted data on municipalities’ centrality (i.e. location of municipalities in relation to urban settlements) grouped in four categories (least central, less central, quite central and central). Municipality age profile was defined by two variables; percent population above 67 years and percent population above 80 years. KOSTRA contains no data regarding the use of municipal rehabilitation services. We therefore decided to include indirect measures, i.e. available healthcare resources in the municipality relevant for rehabilitation, provided > 80% completeness of data; overall staffing (fulltime positions in rehabilitation per 10,000 inhabitants, i.e. home based and nursing home services); nursing home resources were expressed by number of short-term stays divided by number of inhabitants in the municipality, and staffing (available doctor and physiotherapist hour/nursing home patient/week). All variables were divided in quartiles based on the study population.

### Study population

The study population comprised all patients operated due to a hip fracture and reported to the NHFR between 1 January 2011 and 31 December 2012. Follow-up lasted for 1 year after the hip fracture or until death.

### Statistical analysis

Patient characteristics were given as percentages and for the continuous variable as mean, standard deviation (SD), median as well as minimum and maximum values. The resources in the 428 municipalities were listed as percentages, mean and SD. To quantify the association between the age structure of the population and the different municipality resources Pearson bivariate correlation coefficient was calculated.

Mortality was analyzed both as death within 30 days, and death within 1 year after hip fracture surgery. Cox regression model was used to estimate relative risk (RR) and 95% confidence interval (CI) for mortality in different levels of municipality resources with the lowest quartile as reference category. The models were adjusted for patients’ age, sex, ASA, and the municipalities’ centrality.

Each dimension in EQ-5D-3 L was analyzed separately and was dichotomized with the two most favorable levels (no or some problem) as reference, to which the poorest outcome was compared (mobility: bedridden; self-care: unable to wash or dress; usual activity: unable to perform; pain/discomfort: extreme; anxiety/depression: severe). To account for the large proportion of death within 1 year after surgery when analyzing EQ-5D-3 L, invers probability weight (IPW) was estimated from a logistic regression model including variables associated with mortality. To estimate the effect of municipality resources on health-related quality of life, logistic regression analysis with IPW was applied. The estimated odds ratio (OR) was adjusted for age, sex, ASA and centrality. To investigate whether the municipality could explain some of the variation in EQ-5D-3 L or mortality, we performed mixed effect logistic regression with municipality as random effect. Since the intraclass correlation coefficient was in the magnitude of 0.03 and lower, ordinary logistic and cox regression was maintained.

The EQ-VAS score for general health was analyzed in a generalized linear model. The municipality resources were in quartiles; the first quartile was used as reference category, and adjusted for age, sex, ASA and municipalities’ centrality. The results were presented as differences in EQ-VAS score with 95% CI. Analyzes were performed using IBM SPSS Statistics 22 and STATA 13.1; *p*-values lower than 0.05 were considered significant.

## Results

### Study population

The study population comprised 15,757 patients (68.6% women) with a mean age of 80.8 years (SD 11.6 years), and a median ASA score of 3. The median length of index hospital stay was 6 days. On average, patients were readmitted 1.5 (SD 1.2) times during the first year postoperatively; 8.4% of the patients for specific complications after hip fracture surgery, Table 1.

**Table 1:** Patient characteristics

	*n*	%	Mean (SD)	Median	Min-Max
Year					
2011	7841	49.8			
2012	7916	50.2			
Age	15,757		80.8 (11.6)	83.5	6.3–106.0
Sex					
Male	4950	31.4			
Female	10,807	68.6			
ASA classification^a^					
1, A normal healthy patient	767	4.9			
2, A patient with mild systemic disease	5382	34.2			
3, A patient with severe systemic disease	8553	53.0			
4, A patient with severe systemic disease that is a constant threat to life	1053	6.7			
5, A moribund patient who is not expected to survive the operation	13	0.1			
Missing	189	1.2			
Duration hospital stay (hip fracture)	15,515		8.7 (8.2)	6.0	0–131
Patients with readmissions due to complications^b^ 1 year post-surgery	1324	8.4			
All-cause mortality					
Death within 30 days	1360	8.6			
Death within 12 months	3994	25.3			

^*a*^ *ASA-class, 1,2,3,4,5 ASA* American Society of Anesthesiologists

^*b*^ICD-10 codes T81/T84 (e.g. bleeding, mechanical problem, venous thromboembolic complications)

### Municipality characteristics

There were 428 municipalities in Norway in the study period; 35% being characterized as least central, while less, quite and most central were 12, 18 and 35% respectively. Average available doctor time in nursing home increased from 2011 to 2012 by 2.4 min/patient/week (*p* < 0.002). The results showed no differences in available physiotherapist time in nursing home, mean numbers of short-term stays in nursing home, or overall municipal staff time regarding rehabilitation, illustrated in Table 2. The correlations between the age structure of the population and municipality resources were in the magnitude of 0.24 or less.

**Table 2:** Characteristics in the 428 municipalities in Norway by year (Municipality-State-Reporting)

	Year 2011		Year 2012		
	Mean	SD	Mean	SD	*P*-value
Available doctor hour/nursing home patient/week	0.37	0.19	0.41	0.20	0.002
Available physiotherapist hour/nursing home patient/week	0.34	0.26	0.35	0.27	0.414
Fulltime positions in rehabilitation (per 10,000 inhabitants)	12.9	6.2	12.3	6.5	0.243
Short-term stays in nursing homes (by municipality size)	0.62	0.62	0.66	0.65	0.304
Population above 67 year (percentage of inhabitants)	15.7	3.28	16.0	3.31	0.224
Population above 80 year (percentage of inhabitants)	5.5	1.51	5.4	1.47	0.557

### Mortality

All-cause mortality was 8.6% within 30 days, and 25.3% within 12 months postoperatively, Table 1. Mortality was lower in municipalities with the highest overall staff time regarding rehabilitation and marginally higher in municipalities with available nursing home doctor time within the third quartile. Physiotherapist time and number of short-term stays in nursing home was not associated with mortality, Table 3.

**Table 3:** Prevalence and adjusted^a^ relative risk (RR) of mortality by municipality characteristics

	Death within 30 days			Death within 12 months		
Exposure	%	RR	95% CI	%	RR	95% CI
Municipality						
Age profile (% age 67+)						
1 lower quartile	8.6	ref		26.0	ref	
2	8.5	0.98	0.84–1.14	25.6	0.97	0.89–1.06
3	8.6	1.06	0.91–1.25	24.0	0.95	0.87–1.05
4 top quartile	8.8	1.07	0.88–1.29	25.8	1.01	0.91–1.13
Age profile (age 80+)						
1 lower quartile	8.5	ref		26.2	ref	
2	8.3	0.95	0.82–1.11	24.4	**0.89**	**0.82–0.98**
3	8.9	1.11	0.95–1.30	25.5	1.01	0.92–1.11
4 top quartile	8.8	1.07	0.89–1.28	25.5	0.97	0.87–1.08
Nursing homes						
Available doctor time						
1 lower quartile	8.1	ref		24.8	ref	
2	8.8	1.08	0.92–1.27	25.7	1.03	0.94–1.14
3	9.1	**1.20**	**1.03–1.40**	26.0	**1.10**	**1.01–1.21**
4 top quartile	8.5	1.11	0.94–1.30	24.9	1.04	0.95–1.15
Available physiotherapist time						
1 lower quartile	9.0	ref		25.4	ref	
2	8.2	0.93	0.79–1.08	24.8	0.99	0.90–1.08
3	8.7	1.00	0.86–1.17	24.8	1.01	0.92–1.10
4 top quartile	8.5	0.96	0.83–1.12	26.4	1.06	0.97–1.15
Fulltime positions in rehabilitation (per 10.000)						
1 lower quartile	9.0	ref		26.0	ref	
2	8.9	0.97	0.83–1.13	26.4	1.02	0.93–1.11
3	8.7	0.94	0.81–1.09	25.4	0.93	0.85–1.02
4 top quartile	**7.9**	**0.84**	**0.71–0.98**	**23.8**	**0.85**	**0.78–0.94**
Short-term stays in nursing homes by municipality size						
1 lower quartile	8.7	ref		24.9	ref	
2	8.7	1.01	0.88–1.17	25.8	1.03	0.95–1.12
3	8.7	0.98	0.83–1.15	25.3	0.97	0.89–1.07
4 top quartile	8.5	0.93	0.80–1.09	25.5	0.97	0.88–1.06

Bolded data are statistically significant

^a^Adjusted for patients’ age, sex, ASA, and municipalities’ centrality

### Health-related quality of life

Altogether 51% of patients still alive 12 months after hip fracture surgery completed the EQ-5D-3 L questionnaire. The percentage of the population reporting severe problems in EQ-5D-3 L was low before the fracture. After 12 months, a larger percentage was bedridden, unable to wash/dress, and/or unable to perform usual activity; while extreme pain/discomfort and severe anxiety/depression were at about pre-fracture level. An additional figure shows this in more detail [see Additional file 1: Figure S1]. In municipalities with the highest percentage of the population above 80 years, there were lower rates of self-reported anxiety/depression 12 months after surgery, and the estimated mean EQ-VAS score for general health was 3.2 (95% CI: 0.6–5.8) higher compared to the lowest quartile, Table 4. Also in the municipalities with the highest quartile percentage population above 67 years, patients had a higher EQ-VAS score compared to the group with the lowest percentages (3.4; 95% CI: 0.6–6.2), Table 4. Outcomes in municipalities with the highest staffing quotient did not differ from those with the lowest staffing, Table 4. There was no significant association between EQ-VAS and nursing homes resources.

**Table 4:** Health related quality of life 1 year after a hip fracture, prevalence of poorest outcome for the EQ-5D five dimensions by municipality characteristics in quartiles, and estimated odds ratios (OR) with the lowest quartile as reference. Estimated coefficients for general heath measured on a VAS scale with the lowest quartile as reference

	Health related quality of life, EQ-5D-3 L dichotomized															General health	
	Mobility			Self-care			ADL			Pain/discomfort			Anxiety/depression			EQ-5D VAS	
Exposure	%	OR^a^	95% CI	%	OR^a^	95% CI	%	OR^a^	95% CI	%	OR^a^	95% CI	%	OR^a^	95% CI	B ^b^	95% CI
Municipality																	
Age profile (% age 67+)																	
1 lower quartile	4.7	1		11.3	1		23.2	1		6.7	1		5.0	1		0	
2	4.6	1.10	0.69–1.75	11.8	1.23	0.91–1.66	22.4	1.00	0.79–1.26	7.8	1.43	0.98–2.10	4.4	0.96	0.63–1.45	−1.3	−3.8–1.3
3	3.8	1.11	0.62–1.98	9.5	0.97	0.69–1.37	19.1	0.86	0.68–1.09	6.1	1.08	0.72–1.62	4.1	**0.62**	**0.40–0.97**	0.4	−2.2–3.0
4 top quartile	3.8	0.86	0.47–1.57	10.5	0.78	0.54–1.14	22.2	**0.75**	**0.57–1.00**	6.1	1.06	0.70–1.60	3.1	0.55	0.29–1.04	**3.4**	**0.6–6.2**
Age profile (age 80+)																	
1 lower quartile	4.5	1		11.1	1		23.1	1		8.0	1		5.8	1		0	
2	4.8	0.98	0.62–1.54	11.7	1.02	0.76–1.38	22.9	0.86	0.68–1.07	6.6	1.04	0.70–1.55	4.1	0.69	0.46–1.02	0.7	−1.8–3.1
3	3.5	1.27	0.73–2.22	9.4	0.99	0.71–1.39	18.2	**0.77**	**0.60–0.98**	6.4	1.13	0.75–1.70	3.9	0.85	0.52–1.38	1.2	−1.4–3.7
4 top quartile	3.9	0.91	0.53–1.57	10.4	0.92	0.64–1.33	22.6	0.83	0.63–1.09	5.8	0.83	0.56–1.24	2.9	**0.46**	**0.28–0.77**	**3.2**	**0.6–5.8**
Nursing homes																	
Available doctor time																	
1 lower quartile	3.8	1		10.7	1		22.5	1		6.1	1		3.7	1		0	
2	3.1	0.91	0.53–1.55	9.7	0.85	0.61–1.17	**18.7**	**0.74**	**0.58–0.95**	7.1	1.10	0.71–1.70	4.1	1.05	0.65–1.70	2.1	−0.51–4.7
3	4.1	0.92	0.56–1.50	10.6	0.90	0.66–1.22	21.9	0.85	0.67–1.07	6.5	0.95	0.63–1.45	4.5	0.97	0.61–1.53	0.4	−2.03–2.9
4 top quartile	**5.8**	**1.89**	**1.14–3.12**	12.2	1.18	0.85–1.65	23.1	1.02	0.79–1.30	7.2	0.84	0.55–1.29	4.3	1.23	0.70–2.18	−2.5	−5.17–0.3
Available physiotherapist time																	
1 lower quartile	3.8	1		10.2	1		20.5	1		6.1	1		3.5	1		0	
2	3.0	0.79	0.47–1.32	10.6	0.855	0.62–1.15	22.6	0.94	0.75–1.18	7.5	1.20	0.80–1.80	4.8	1.06	0.67–1.68	0.1	−2.1–2.4
3	4.3	1.27	0.76–2.13	10.3	0.98	0.72–1.35	20.5	0.90	0.71–1.15	6.8	1.00	0.68–1.48	3.9	0.85	0.52–1.39	−1.8	−4.2–0.6
4 top quartile	**5.8**	**1.59**	**1.00–2.53**	12.1	1.14	0.85–1.54	23.1	1.07	0.85–1.34	6.5	1.11	0.73–1.70	4.6	1.23	0.79–1.91	−2.4	−4.8–0.1
Fulltime positions in rehabilitation (per 10,000)																	
1 lower quartile	4.3	1		10.1	1		20.5	1		6.7	1		4.4	1		0	
2	3.1	0.64	0.35–1.17	8.8	0.90	0.63–1.29	21.0	1.06	0.83–1.35	6.2	0.85	0.54–1.33	4.3	0.75	0.44–1.27	1.3	−1.3–3.8
3	5.0	0.96	0.62–1.50	11.9	1.12	0.84–1.51	23.0	1.01	0.81–1.27	7.2	0.98	0.66–1.46	4.4	0.86	0.54–1.38	0.7	−1.4–2.9
4 top quartile	4.7	1.04	0.62–1.75	12.0	1.06	0.77–1.48	22.2	1.01	0.79–1.28	6.7	1.05	0.67–1.63	3.6	0.81	0.46–1.42	0.2	−2.2–2.6
Short-term stays in inursing homes by municipality size																	
1 lower quartile	3.7	1		10.6	1		21.2	1		7.5	1		3.9	1		0	
2	4.2	1.38	0.84–2.27	11.1	1.19	0.88–1.62	21.8	1.01	0.81–1.27	5.9	0.77	0.52–1.15	4.6	1.01	0.67–1.52	−0.8	−3.2–1.7
3	5.0	1.39	0.87–2.23	11.0	1.13	0.82–1.56	22.0	0.99	0.78–1.27	6.4	0.95	0.62–1.44	4.3	1.38	0.86–2.22	−1.8	−4.3–0.7
4 top quartile	3.9	1.08	0.67–1.74	10.4	0.92	0.68–1.25	21.5	0.87	0.69–1.09	7.1	1.03	0.72–1.47	3.7	0.81	0.52–1.25	0.0	−2.3–2.3

Invers probability weight was applied in the analyses to account for the large proportion of death within 1 year after surgery

Bolded data are statistically significant

^a^Adjusted for patients’ age, sex, ASA, and municipalities’ centrality

^b^Estimated marginal means, adjusted for patients’ age, sex, ASA, and municipalities’ centrality

## Discussion

### Main results

This study showed only a minor impact of municipal resources on survival and quality of life through the first year after a hip fracture. Mortality was lower in municipalities with most overall staff time regarding rehabilitation, but marginally higher in municipalities with relatively more available nursing home doctor time. Health-related quality of life, in terms of overall satisfaction and five distinct dimensions, was not associated with municipal resources in the studied sample. A high percentage of the population above 80 years, was associated with lower rates of self-reported anxiety/depression 12 months after surgery, as well as higher scores for general health.

### Methodological considerations

Whereas the large sample size is the major strength of our study, the sparse amount of information available per individual is the most important limitation. In this nationwide study, data from two quality health registries provided valid and reliable measures for demographics, hip fracture, hospital readmission and death. The NHFR comprises 90% of all hip fracture operations in Norway [19]; we assume that underreporting is non-differential, with no impact on the results. Data on health-related quality of life was available for half of the population alive after 12 months; thus, we cannot rule out reporting bias in either direction. At present, there is a lack of reliable municipality-based clinical data at the patient level (e.g. diagnoses, functional ability), and contribution of health personnel (e.g. profession, competence, care level). Data from Municipal-State-reporting of community services relevant for rehabilitation after hip fracture are the best proxy measures available; however, they represent crude measures and sources of error.

Hospital based data of factors such as fracture type, mode of operation and inpatient treatment may influence patients’ potential for rehabilitation but were not included in the present study. The largest municipalities (high centrality) in Norway are hosting several hospitals, while several smaller municipalities (lower centrality) are covered by one hospital. Mixed effect logistic regression with municipality as random effect revealed that municipality could not explain the variation in EQ-5D-3 L or mortality.

Both organizational factors in the health care system (mainly public in Norway), and available rehabilitation options must be considered in transferring our results to other countries.

### Discussion of results

#### Study population

The study population comprised older people with concomitant chronic disease. A previous study including 1010 patients hospitalized for hip fracture in one hospital in south-eastern Norway from 2007 to 2009 revealed that half of the patients were discharged to municipal rehabilitation in nursing home (short-term stay, 33%), or home (17%) [9]. A fourth of patients was returned to long-term care, and 17% referred to a rehabilitation centre within specialist care [9]. The corresponding percentages could not be identified in the present study, which may be explained by the fact that specialised rehabilitation centres are less available in other regions. Thus, in our study, we assume that the majority of patients were eligible for rehabilitation in primary health care in the municipality. It may be expected that long-term care patients had severe functional impairment prior to the hip fracture, with low potential for rehabilitation.

#### Municipality resources

Average available doctor time in nursing home increased marginally during the study period (by about two minutes per patient per week), while the other measures of staffing did not change. Though the Coordination reform, the municipalities were given increased responsibility for rehabilitation and community-based treatment for severely ill patients with comorbidity. Based on these demands, stability in staffing is expected to reduce availability of services to patients with hip fractures, as they compete with other patient groups for municipal rehabilitation resources. Based on routine municipality data today, there is no possibility of examining individual patient pathways after hospital discharge, or determine the effect of invested resources in care and rehabilitation.

#### Mortality according to staffing

All-cause mortality after 1 year was in line with previous studies, supporting the quality of health register data used in this study [20, 21]. Our finding of lower mortality in municipalities with more overall staff time regarding rehabilitation seems reasonable. We consider this variable to be rather specific for the purpose of our study. However, lack of community based individual data does not provide knowledge of the category of services, (i.e. patients’ home, nursing home or dedicated municipal rehabilitation institutions). Associations between available doctor and physiotherapist time and mortality were inconsistent; therefore, the marginally higher mortality with increasing doctor time may be explained by several factors. Because of the crude measures used, high staffing may still be too low to produce measurable effects. Increased staffing may also be a consequence of health needs, i.e. poor health status may be a confounder in the relationship between staffing of doctors and patient mortality. Knowledge of local resources in the communities may have influenced hospital practice, i.e. early discharge is more likely in presence of available resources.

A number of other factors may also have influenced the relationship between staffing and the outcome variables. In the community databases, there is no information about the competence of doctors and physiotherapists, and the weekly number of hours employed in the nursing homes. Traditionally many of the positions for doctors have been minor part time positions for general practitioners, which do not facilitate in-depth knowledge within relevant fields. For other staff there is no information about profession or formal education.

#### Health-related quality of life

Twelve months after hip fracture surgery, the percentage of the population with severe pain and mood symptoms (anxiety/depression) was largely at pre-fracture level, while severe functional impairment (bedridden, unable to wash/dress or perform usual activities) was increased. However, an increase in level of disability is not unexpected based on age-group and ASA score [3]. Outcomes in municipalities with more staffing (top quartile) were not better; however associations between available staff time and health-related quality of life were inconsistent and should therefore be interpreted with cause. The consistent finding of lower rates of self-reported anxiety/depression 12 months after surgery in municipalities with a higher proportion of the population above 80 years, may suggest that these communities provide better living conditions for older people in general.

#### Implications for practice and research

Stability of staffing contradicts the major goals of the Coordination reform, and may contribute to the lack of association between patient measures and other rehabilitation resources. The present study highlights the need for more detailed community based data to monitor ordinary services on a continuous basis, allowing to examine studies of effect based on reliable measures and identified goals for health and functional ability. In addition, there is a need for more detailed information of profession and competence of staff, as well as the number of full time and major positions. Such measures may contribute to ensure quality and design of sustainable services, as well as adequate resources for the anticipated increase in hip fractures. To achieve this, systematic collaboration between services and research environments is suggested, as well as arenas for communication with service users.

## Conclusions

The study revealed no substantial impact of municipal resources on survival and health-related quality of life through the first year after a hip fracture, based on crude measures of municipal characteristics. The context of this study highlights a need to monitor use of resources and health outcomes over time, through reliable and valid patient and personnel measures, and also including variables related to coordination of services. Quality databases for establishing national figures at the municipality level, and corresponding procedures to comply with privacy and ethical guidelines are essential for planning and evaluation. General practitioners have an important role in coordinating health services for individual patients’ and identifying relevant variables that can be monitored through clinical practice.

## Additional file

Additional file 1: Figure S1. Health-related quality of life (EQ-5D-3 L); patient share with poorest outcomes. Legend: Preoperative (blue columns) and 12 months postoperative (red columns). (PDF 167 kb)

## Abbreviations

ASA: American Society of Anesthesiologists
NHFR: Norwegian Hip Fracture Register
NPR: Norwegian Patient Register
VAS: Visual analog scale
