# Supplementary figures and images for: Municipal resources and patient outcomes through the first year after a hip fracture
Source: BMC Health Serv Res. 2017 Feb 16;17:144. doi: 10.1186/s12913-017-2087-5 (PMC5314693; doi:10.1186/s12913-017-2087-5)

## Additional file 1

### Health-related quality of life (EQ-5D-3L); patient share with poorest outcomes

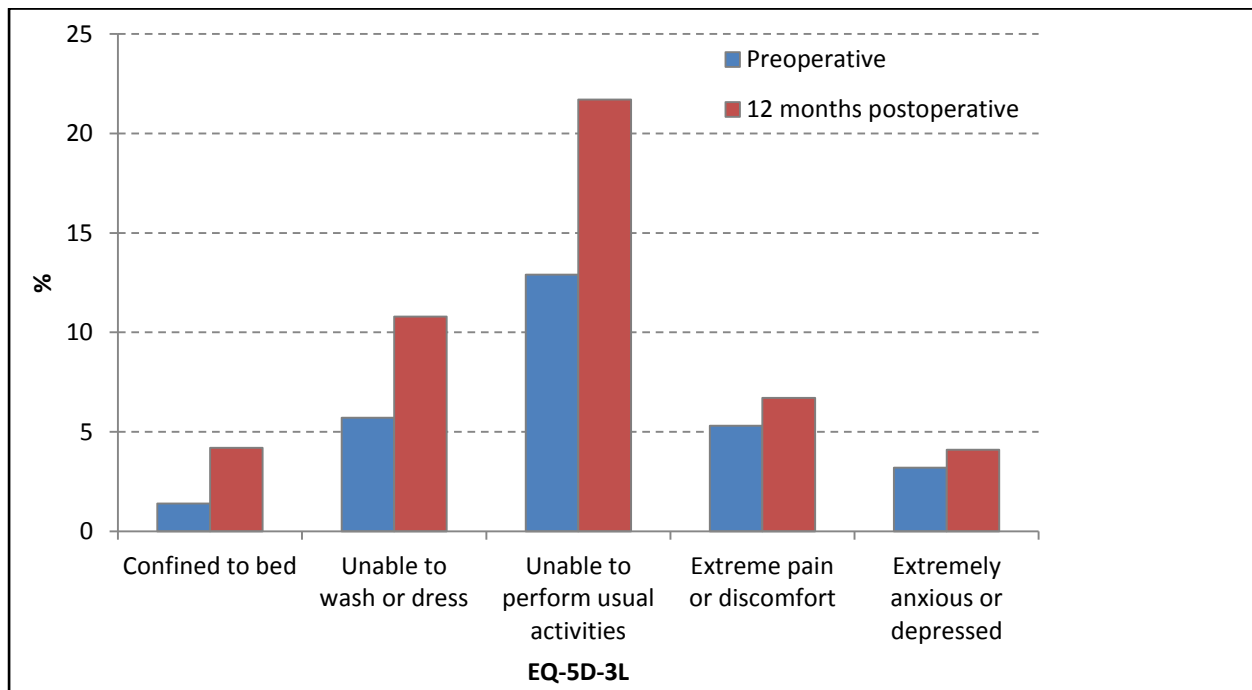

Supplement: Additional file 1: Figure S1. — Health-related quality of life (EQ-5D-3 L); patient share with poorest outcomes. Legend: Preoperative (blue columns) and 12 months postoperative (red columns). (PDF 167 kb) [file 12913_2017_2087_MOESM1_ESM.pdf]
